# OP22 Productivity Benefits Of Modifying Cardiovascular Disease Risk In Type 2 Diabetes In Australia: A 10-Year Prediction Dynamic Model

**DOI:** 10.1017/S0266462324000850

**Published:** 2025-01-07

**Authors:** Dina Abushanab, Daoud Al-Badriyeh, Clara Marquina, Jedidiah I Morton, Melanie Lloyd, Ella Zomer, Stella Talic, Danny Liew, Zanfina Ademi

## Abstract

**Introduction:**

Diabetes has been shown to influence the individual’s work productivity in terms of both presenteeism (reduced productivity while at work) and absenteeism (absence from work because of illness). We sought to estimate the potential productivity gains associated with the modification of cardiovascular disease (CVD) risk in type 2 diabetes (T2D) over the next 10 years in Australia, from 2023 to 2032.

**Methods:**

Dynamic models were structured to estimate productivity-adjusted life years (PALYs) lived by Australians aged 20 to 69 years. The model simulation was first undertaken assuming currently expected trends in the incidence of myocardial infarction (MI) and stroke in T2D (original cohort), as calculated using the 2013 PCE-ASCVD algorithm. Subsequent models were then re-simulated using hypothetical scenarios that reflected the potential benefits of CVD reduction using published trials. The model was also repeated assuming that the original cohort has no CVD. Differences in PALYs lived by the “original cohort” and the different cohorts with reduced CVD risk reflected the PALYs gained. Sensitivity analyses were conducted.

**Results:**

Using data from published studies, the model assumed a reduction of 50 percent in systolic blood pressure (SBP), a reduction of 50 percent in smoking, 50 percent increase in high-density lipoprotein cholesterol (HDL), and a reduction of 35 percent in incidence of T2D. Over the working lifetime, from 2023 to 2032, reducing SBP, smoking, and incidence of T2D led to the gain of 140,105, 333,127, and 998,805 PALYs, respectively. Further, increasing HDL and assuming the original cohort with T2D has no CVD are expected to lead to the gain of 71,623 and 889,455 PALYs, respectively. Sensitivity analyses confirmed the robustness of study findings.

**Conclusions:**

The impact of CVD as a complication from T2D on work productivity is significant. Screening and prevention strategies tailored early in life are likely to exert a positive impact on health and work productivity.

